# Sex-biased genes and metabolites explain morphologically sexual dimorphism and reproductive costs in Salix paraplesia catkins

**DOI:** 10.1038/s41438-021-00566-3

**Published:** 2021-06-01

**Authors:** Zeyu Cai, Congcong Yang, Jun Liao, Haifeng Song, Sheng Zhang

**Affiliations:** 1 Key Laboratory of Mountain Surface Processes and Ecological Regulation, Institute of Mountain Hazards and Environment, Chinese Academy of Sciences, Chengdu, China; 2 University of Chinese Academy of Sciences, Beijing, China; 3 Key Laboratory of Bio-Resource and Eco-Environment of Ministry of Education, College of Life Sciences, Sichuan University, Chengdu, China; 4 College of Geography and Tourism, Chongqing Normal University, Chongqing, China

**Keywords:** Plant ecology, Molecular ecology

## Abstract

Dioecious species evolved from species with monomorphic sex systems in order to achieve overall fitness gains by separating male and female functions. As reproductive organs, unisexual flowers have different reproductive roles and exhibit conspicuous sexual dimorphism. To date, little is known about the temporal variations in and molecular mechanisms underlying the morphology and reproductive costs of dioecious flowers. We investigated male and female flowers of *Salix paraplesia* in three flowering stages before pollination (the early, blooming and late stages) via transcriptional sequencing as well as metabolite content and phenotypic analysis. We found that a large number of sex-biased genes, rather than sex-limited genes, were responsible for sexual dimorphism in *S. paraplesia* flowers and that the variation in gene expression in male flowers intensified this situation throughout flower development. The temporal dynamics of sex-biased genes derived from changes in reproductive function during the different flowering stages. Sexually differentiated metabolites related to respiration and flavonoid biosynthesis exhibited the same bias directions as the sex-biased genes. These sex-biased genes were involved mainly in signal transduction, photosynthesis, respiration, cell proliferation, phytochrome biosynthesis, and phenol metabolism; therefore, they resulted in more biomass accumulation and higher energy consumption in male catkins. Our results indicated that sex-biased gene expression in *S. paraplesia* flowers is associated with different reproductive investments in unisexual flowers; male flowers require a greater reproductive investment to meet their higher biomass accumulation and energy consumption needs.

## Introduction

Sexual dimorphism in dioecious plants refers to sexual differences in primary and secondary sex characteristics^1,2^. Such divergences between males and females are the results of sexual selection for traits that influence individual fitness during the evolution process^3^. Selective pressure may lead to sexual antagonism, in which traits are beneficial to one sex but harmful to another^4–6^. It is important to distinguish between sexual dimorphic traits caused by primary genetic changes that lead to the loss of male or female functions and secondary changes in adaptive states. As a direct consequence of the loss of female or male reproductive function during the evolution of dioecy, primary sexual dimorphism occurs in reproductive organs and is reflected in the androecium and gynoecium. The different roles of flowers in mating, during which males spread pollen and females capture it, result in the development of different flower phenotypes and reproductive production.

At the individual level, the reproductive investment of females, which is responsible for seed production, is usually greater than that of males, which is responsible for pollen generation^7^. However, determining the reproductive allocation within a population is complicated because (1) in some wind-pollinated species, males shed their flowers throughout the mating season to increase their pollination success rate^8^ and (2) the numbers of male flowers and pollinated female flowers are random, resulting in varying reproductive allocation patterns between males and females at the whole-plant level^9,10^. Studies on *Silene latifolia* have noted that males exhibited higher reproductive costs because they invest more biomass in reproduction^11^. Ultimately, female-biased sex ratios are rarer than male-biased sex ratios in dioecious species, and the dominant status of the male-biased sex ratio is the consequence of reproductive sexual dimorphism^12^.

Unlike animals, plants show fewer sex characteristics during their juvenile period. As there are no distinctly sexual traits in the morphology or physiology of vegetative tissue, secondary sexual dimorphism is hard to observe, and the sex of dioecious individuals is difficult to distinguish until flowering. At the genetic level, the genomes of dioecious species lack sex-determination regions or have only a small number of different genetic loci on sex chromosomes. Sexual dimorphism is derived mostly from differences in gene dosages and gene regulation rather than sex-limited gene expression^13–18^. The expression patterns of these genes differ between unisexual individuals and are so-called sex-biased genes. Because sex-biased genes are more common in reproductive tissues (flowers) than in vegetative tissues (leaves and roots), reproductive tissues better reflect information about fitness conflicts than vegetative tissues in dioecious plants^19,20^. There have been many studies on sexual dimorphism in reproductive traits^1^, *e.g*., flowering phenology, flower number^11^, flower color^20^, flower size^21,22^, nutrient content^11^, and flower defense ability, but few studies have provided information about the genetic basis of secondary sexual dimorphism in flowers. All these sexual dimorphisms in flower phenotypic traits result from differential gene expression and the metabolites involved in biological processes. Flowering phenology, catkin morphology, and another differentiation are tightly correlated with several phytohormones^23,24^. Catkin color is correlated with pigments in the upper epidermal cells, such as chlorophyll, carotenoids, flavonoids, and alkaloids^25–27^. The morphogenesis of flowers is a dynamic program, and unisexual flowers of dioecious plants play different roles during their developmental stages^28^. Thus, genes probably exhibit temporal dynamics of sex-biased expression patterns during flower development. To date, few studies have focused on the variation in the secondary sexual dimorphism of flowers and its genetic basis throughout florescence.

Willows are a type of woody angiosperm, and there are approximately 400 willow species worldwide. Willows evolved dioecy 45 Ma ago. Thus, willows are suitable for investigations of sexual dimorphism^29–32^. There have been abundant studies about the biased sex ratios of this genus, and female bias is more common in *Salix* populations than in other species^33–35^. *Salix paraplesia* Schneid., a pioneer tree species used in alpine ecosystem conservation, is widely distributed on the eastern Tibetan Plateau, China^36^. As a dioecious plant, *S. paraplesia* shows a female-biased sex ratio as well as sexually differentiated responses to abiotic stresses^37^. We assumed that these ecological and physiological differences could be attributed to sex-specific reproductive investments. To clarify the sexual dimorphism of *S. paraplesia* and the potential relationship between these differences and reproductive investments, we explored unisexual *S. paraplesia* flowers at the phenotypic, metabolic, and transcriptomic levels in three flowering stages before pollination. In this study, we focused on two objectives: (1) characterizing the variations in sexual dimorphism among the different flowering stages and (2) determining the reproductive costs of sexual dimorphism.

## Material and methods

### Collection site and sampling

Male and female flowers of *S. paraplesia* were collected from Wanglang National Nature Reserve (32°49′N–33°02′N, 103°55′E–104°10′E) from March to April 2018 on three dates (March 9th, March 29th, and April 18th) before the male flowers withered and the female flowers fruited. This region is part of the monsoon climate zone and has distinct dry (November to April) and wet (May to October) seasons. The air temperature during the growing season at the sampling site was 10–23 °C, and the mean annual precipitation was 801–825 mm^37^. The three sampling dates represented three flowering stages of *S. paraplesia* (the early stage, blooming stage, and late stage) according to Biologishe Bun-desanstalt, Bundessortenamt, and Chemical Industry^38^. The study was performed in three plots (20 × 20 m) at an altitude of 2600 m. The plots were spaced at >100 m intervals. Five adult male and five adult female trees that were over 30 years old and exhibited similar morphological features were selected in each plot. The collected flower samples (approximately 100 catkins from each tree) were packaged in aluminum foil, labeled by sex and date, and immediately frozen in liquid nitrogen. They were then stored at -80 °C until use. Other flower samples were scanned for morphological analysis and then oven-dried and ground for the nutrient and physiological analyses.

### Morphology, biomass, and nutrient analysis

After collection, the fresh flower samples were scanned by a CanoScan LiDE300 (Canon CHN, Beijing, China). The length and projected area of each flower were calculated by ImageJ 1.8.0_ sexual dimorphism flower phenology2 (https://imagej.nih.gov/ij/download.html). For the dry weight measurement, the flowers were dried to a constant weight (105 °C for 30 min and then 70 °C for 48 h) and weighed. The dried flowers were ground to a powder for the nutrient analysis. For the carbon (C) and nitrogen (N) concentration measurements, 500 mg of dried flower samples were analyzed using a Vario MACRO cube C/N-elemental analyzer (Elementar Analysensyteme GmbH, Hanau, Germany). For the phosphorus (P) concentration measurements, the dried flower samples (500 mg) were digested in sulfuric acid and measured using the vanadium–molybdenum–yellow photometric method^39^. The nutrient content per catkin was calculated by multiplying the nutrient concentration by the arithmetic average of the dry weight. For the chlorophyll content measurements, 500 mg flower samples were soaked in 8 mL extraction solution (4 mL 100% acetone + 4 mL 100% ethanol) for 24 h in the dark. The concentrations of chlorophyll a and b were determined by Arnon’s method^40^. SPSS Statistics 26 (IBM, Armonk, USA) was used for statistical analysis. The column and violin diagram were constructed by ggplot2 (version 3.3.2) in R 4.0.2 (https://mirrors.ustc.edu.cn/CRAN/)^41,42^.

### Illumina sequencing and data processing

We used three biological replicates from each group of *S. paraplesia* flowers for RNA sequencing. The RNA of the flower samples was extracted using the RNAprep Pure Plant Kit (TIANGEN, Beijing, China). The cDNA library was generated from the total RNA using the NEBNext UltraTM RNA Library Prep Kit for Illumina (NEB, Ipswich, USA) and purified using the AMPure XP system (Beckman Coulter, Beverly, USA). The quality and quantity of the cDNA library were determined using an Agilent 2100 bioanalyzer (Agilent, Santa Clara, CA, USA). Clustering of the index-coded samples was performed on a cBot Cluster Generation System using the TruSeq PE Cluster Kit v3-cBot-HS (Illumina, San Diego, CA, USA) according to the manufacturer’s instructions. After cluster generation, the prepared libraries were sequenced on an Illumina NovaSeq platform, and 150-bp paired-end reads were generated. Clean data (clean reads) were obtained by removing reads containing adapters, reads containing poly-N sequences and low-quality reads from the raw data. At the same time, the Q20, Q30, and GC contents of the clean data were calculated. All downstream analyses were based on clean, high-quality data. We compared the clean reads with the reference genome (*Salix purpurea v5.1*, https://genome.jgi.doe.gov/portal/pages/dynamicOrganismDownload.jsf?organism=Spurpurea) using Hisat2 v2.0.5 (http://ccb.jhu.edu/software/hisat2/faq.shtml). Feature Counts v1.5.0-p3 was used to count the read numbers mapped to each gene (http://subread.sourceforge.net). Finally, the fragments per kilobase million (FPKM) of each gene was calculated based on the length and the read count mapped to the gene.

### Differentially expressed gene analysis

Differential expression analyses between groups (with three biological replicates per group) were performed using the DESeq2 R package (1.16.1)^43^. Genes with a corrected *P-value* < 0.05 as identified by DESeq2 were considered differentially expressed genes (DEGs). We defined those genes expressed (FPKM > 1) in only single-sex groups as sex-limited genes. The DEGs between male and female groups from the same flowering stage were defined as sex-biased genes. For Gene Ontology (GO) annotation and KEGG annotation, the NCBI and KEGG databases contained the annotation for the *Populus trichocarpa* genome but lacked the annotation for the *Salix purpurea* genome. Because *Populus* and *Salix* are both in the Salicaceae family, their chromosomal structures are highly similar^44,45^. First, we compared the *Salix purpurea v5.1* genome with the *Populus trichocarpa v3.1* genome, and the BLASTN algorithm was applied for analysis with an E value cutoff of 10^-10^. Then, we used the Entrez ID of homologous *Populus trichocarpa* genes for downstream analysis. GO enrichment analysis and KEGG enrichment analysis of the DEGs were conducted by the clusterProfiler R package (3.16.1)^46^, in which the gene length bias was corrected. GO terms with corrected *P* values (*P*_*adj*_) < 0.05 were considered significantly enriched in DEGs. KEGG pathways with *P* values less than 0.05 were considered significantly enriched. The differentially expressed genes were also annotated to pathways by MapMan v3.6.0^47^. The sequences of all genes of *S. paraplesia* were annotated to the plaBi database using Mercator v3.6^48^ to generate the mapping file for MapMan analysis (https://www.plabipd.de/portal/web/guest/mercator-sequence-annotation). Venn diagrams were produced by VENNY 2.1 (https://bioinfogp.cnb.csic.es/tools/venny/index.html)^49^. The principal component analysis (PCA) score diagram, bubble diagrams, and cluster diagrams were produced by the R packages ggplot2 (version 3.3.2) and pheatmaps, respectively^42,50^.

### Validation of transcriptomic profiles by RT-qPCR

To confirm the reliability of the RNA-seq profile, we selected 19 DEGs involved in different pathways for analysis with real-time quantitative PCR. RNA was extracted from flower samples using a Plant RNA Extraction Kit (Biofit Biotechnologies, Chengdu, China). The quality and integrity of the total RNA were verified using the Gel Imager System Gel Doc XR + (Bio-Rad, USA) and a OneRop OD1000 + spectrophotometer (Wins, Nanjing, China). Then, cDNA was obtained using Hifair® III 1st Strand cDNA Synthesis SuperMix for qPCR (gDNA Digester Plus) (Yeasen, Shanghai, China). The coding sequence of the genes was obtained from Phytozome v13. The primers of the genes were designed using the NCBI Primer-Blast tool (Table S4). Because the known sequence of the 18 S rRNA of *S. purpurea* was incomplete, we selected 5.8 S rRNA as the reference gene. Quantitative reactions were conducted on a Bio-Rad CFX96 Touch Real-Time PCR system (Bio-Rad, Hercules, CA, USA) using 2X SG Fast qPCR Master Mix (Sangon Biotech, Shanghai, China). qPCR was performed according to the following protocol: one cycle for 3 min at 95 °C, followed by 40 cycles at 95 °C for 3 s, and holding at 60 °C for 30 s. The relative expression of the genes was calculated with Livak’s method^51^.

### Nontarget metabolic relative quantification analysis

We analyzed five biological replicates from each group, and each replicate contained 200 mg of fresh flower samples. In addition, fresh flower samples from all groups were fully mixed, and six biological replicates of the mixed samples were performed as quality controls (QCs). First, 50 ± 1 mg samples were placed into 2 mL tubes and extracted with 450 μL extraction liquid (V_Methanol_: V_H2O_ = 3:1). For the internal standard, 10 μL of L-2-chlorophenylalanine (1 mg mL^-1^ stock in dH_2_O) was added and vortexed for 30 s. The mixture was homogenized in a ball mill for 4 min at 45 Hz and then ultrasonically treated for 5 min while incubated in ice water. Then, all samples were centrifuged for 15 min at 12000 rpm and 4 °C. After centrifugation, 300 μL of the supernatant was transferred into a fresh 1.5 mL tube. Then, 70 μL portions of the mixture from each sample were mixed to form the QC sample. The supernatants were dried completely in a vacuum concentrator without heating. Methoxyamination hydrochloride (130 μL, 20 mg mL^-1^ in pyridine) was added to the samples, and the samples were incubated for 30 min at 80 °C. Then, 150 μL of BSTFA reagent (1% TMCS, v/v) (REGIS Technologies, Morton Grove, USA) was added to the sample aliquots and incubated for 1.5 h at 70 °C. Finally, fatty acid methyl esters (Dr. Ehrenstorfer, Augsburg, Germany) were added to the QC sample as it cooled to room temperature. All samples were analyzed with a gas chromatography system coupled with a Pegasus HT time-of-flight mass spectrometer (GC-TOF-MS).

GC-TOF-MS analysis was performed using an Agilent 7890 GC system coupled with a Pegasus HT time-of-flight mass spectrometer (Agilent, Santa Clara, CA, USA). The GC system utilized a DB-5 MS capillary column (J&W Scientific, Folsom, CA, USA). A 1 μL aliquot of the sample was injected in splitless mode. Helium was used as the carrier gas, the front inlet purge flow was 3 mL min^−1^, and the gas flow rate through the column was 1 mL min^−1^. The initial temperature was held at 50 °C for 1 min, raised to 310 °C at a rate of 10 °C min^−1^, and then held for 8 min at 310 °C. The injection, transfer line, and ion source temperatures were 280, 280, and 250 °C, respectively. The energy was -70 eV in electron impact mode. The mass spectrometry data were acquired in full-scan mode with an m/z range of 50–500 at a rate of 12.5 spectra per second after a solvent delay of 6.1 min.

The Chroma TOF 4.3X (LECO, St. Joseph, USA) and LECO-Fiehn Rtx5 databases were used for raw peak extraction, data baseline filtering, baseline calibration, peak alignment, deconvolution analysis, peak identification, and integration of the peak area. Mass spectrum matching and retention index matching were considered in metabolite identification. Peaks detected in <50% of QC samples or with an RSD > 30% in QC samples were removed. In total, 692 peaks were retained, and 256 metabolites were identified. We normalized the peak data using ribitol as the internal standard. The metabolites in the compared groups were identified as being significantly different if they met the criteria of having variable importance in projection (VIP) values > 1 in the orthogonal partial least squares discrimination analysis (OPLS-DA) model and *P* < 0.05 in the independent-samples *t*-test. PCA and OPLS-DA analyses of the samples were conducted using SIMCA 14.1 (Umetrics, Goettingen, Germany), and *t*-tests were conducted using SPSS Statistics 26 (IBM, Armonk, USA). KEGG annotation and enrichment analysis of the metabolites were conducted using MBROLE 2.0^52^, and the background set was *P. trichocarpa* (black cottonwood).

Venn diagrams for the different metabolites were generated with VENNY 2.1 (https://bioinfogp.cnb.csic.es/tools/venny/index.html)^49^. The cluster diagrams were generated by the R package pheatmaps^50^. The bubble diagrams were generated by the R package ggplot2 (version 3.3.2)^42^.

## Results

### Dimorphism in flower morphology and nutrient levels

**Fig. 1:**
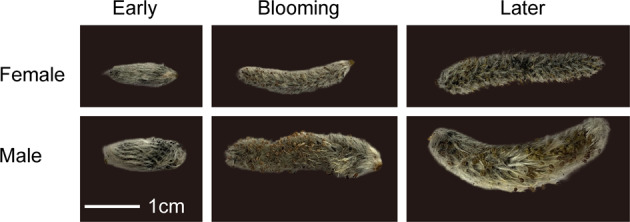
Male (bottom) and female (top) flowers of *S. paraplesia* in the early, blooming, and late flowering stages (from left to right). The white line represents 1 cm

**Fig. 2:**
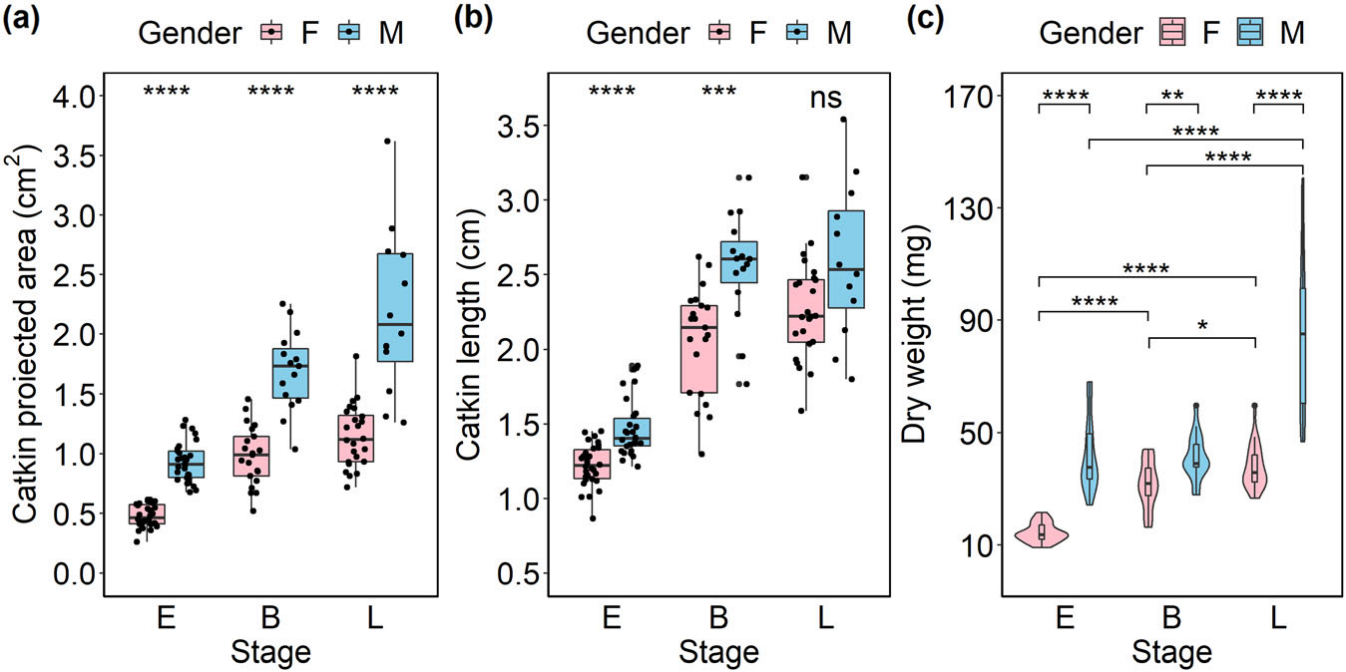
Morphology and biomass of *S*. *Paraplesia* flowers in three stages. **a** Catkin projected area of male (blue) and female (pink) flowers. **b** Catkin length of male (blue) and female (pink) flowers. **c** Biomass of male (blue) and female (pink) *S. paraplesia* flowers. E, early stage; B, blooming stage; L, late stage; ns, no significant difference between groups according to the Mann–Whitney U test; *, *P* < 0.05; **, *P* < 0.01; ***, *P* < 0.001; ****, *P* < 0.0001

Compared with female flowers, male flowers of *S. paraplesia* had longer catkins and larger projected areas in all three flowering stages (Figs. 1 and 2a, b). However, there were no significant differences in catkin length or projected area between the blooming and late flowering stages within each sex (Table S1). Additionally, we found a significant sexual difference in the catkin dry weight in the three flowering stages (Fig. 2c). Interestingly, there were sexual differences in biomass accumulation with flower development. Unlike female catkins, male catkins exhibited no significant difference in biomass between the early and blooming stages, suggesting that the reproductive investment in male flowers was probably used for energy consumption rather than for biomass accumulation.

**Fig. 3:**
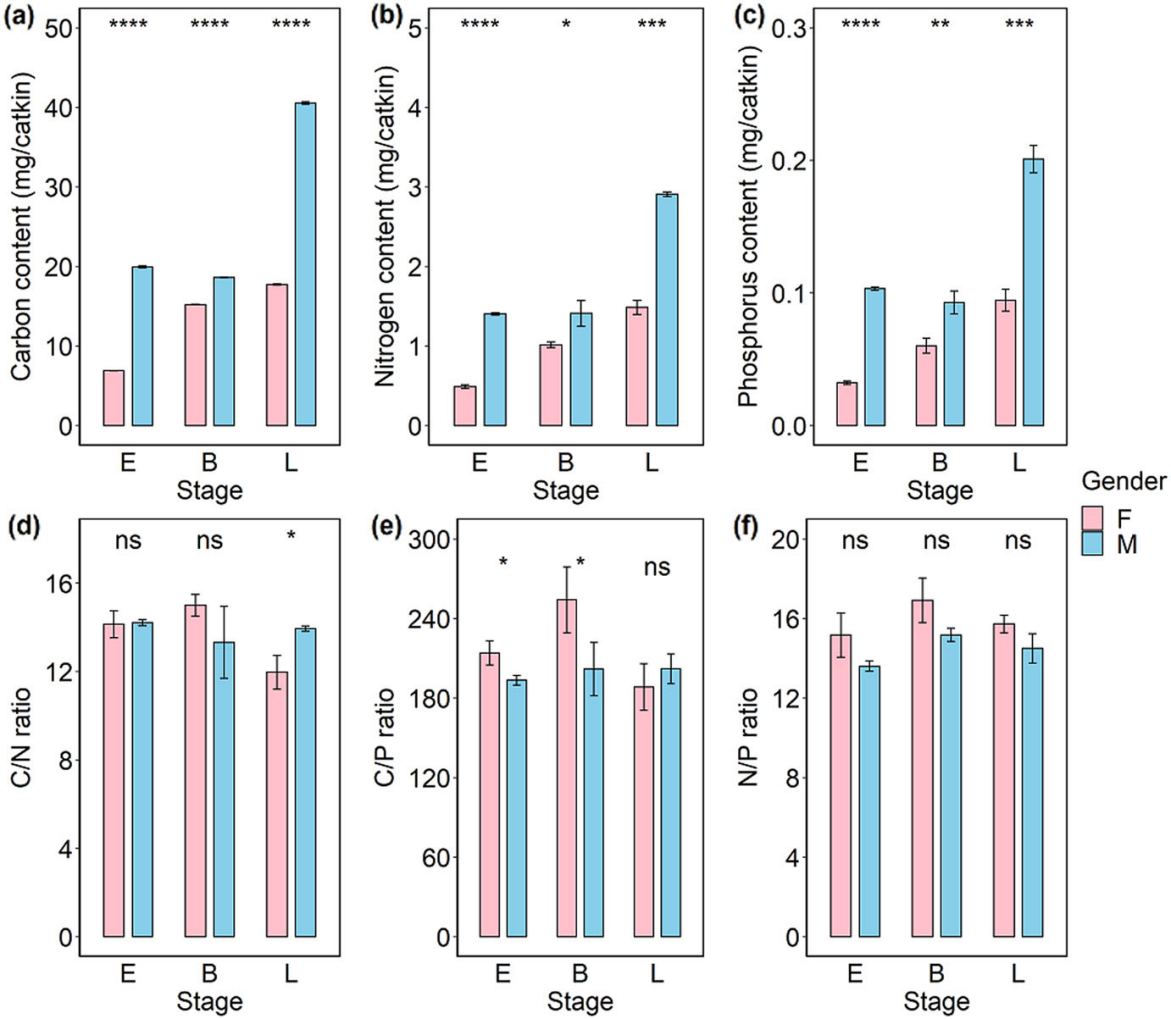
The content and stoichiometry of nutrients of *S. paraplesia* flowers in three stages. **a** Carbon content of catkins. **b** Nitrogen content of per catkin. **c** Phosphorus content of catkins. **d** Carbon to Nitrogen ratio (C/N ratio) of catkins. **e** Carbon to Phosphorus ratio (C/P ratio) of catkins. **f** Nitrogen to Phosphorus ratio (N/P ratio) of catkins. Pink columns, female flowers; Blue columns, male flowers; E early stage; B blooming stage; L late stage; ns no significant difference between compared groups according to a *t*-test; *P < 0.05; **P < 0.01; ***P < 0.001; *P* < 0.0001

The results of the analyses of nutrient concentrations and flower biomass together suggest that male individuals may invest more nutrients than females into flowers before pollination. Male flowers exhibited significantly higher C, N, and P contents than female flowers in all three flowering stages (Fig. 3a–c). Additionally, at the early and blooming stages, the C:P ratios were higher in female flowers, while the C:N ratios were higher in male flowers. Female flowers exhibited higher N:P ratios, although the difference was not statistically significant (Fig. 3d–f).

### Dimorphism in gene expression patterns in flowers

**Fig. 4:**
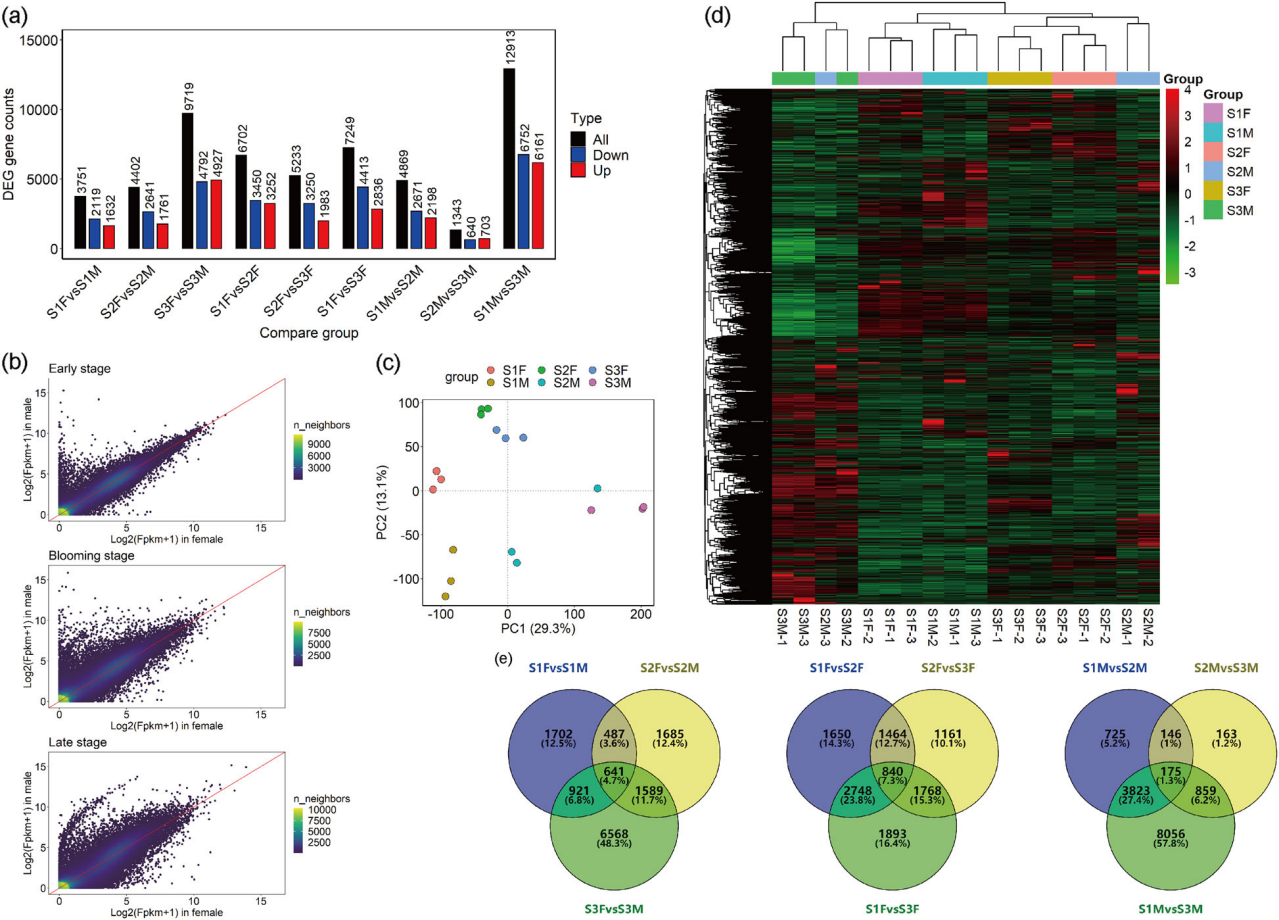
Transcriptome profiles of male and female *S. paraplesia* flowers in three stages. S1, early stage; S2, blooming stage; S3, late stage; F, female; M, male. **a** Count of DEGs among each group of flower samples. For XvsY, black columns represent all DEGs between X and Y; blue columns represent upregulated DEGs in Y; red columns represent upregulated DEGs in X. **b** Expression pattern of genes in male and female flowers in the early, blooming, and late flowering stages. **c** PCA of gene expression patterns in flowers. PC1, the first principal component; PC2, the second principal component. **d** Heatmap of gene expression clustering analysis. The color palette represents the variation in gene expression among flower samples. Rows represent genes, columns represent flower samples. Red represents high gene expression, and the green represents low gene expression. **e** Venn diagram of DEGs in the compared flower groups. The number in each circle represents the number of DEGs expressed in this stage. Numbers in each overlapping part represent DEGs that were coexpressed in multiple stages

On average, 7.78 G transcriptome data were obtained from the samples of *S. paraplesia* from the three flowering stages. The error rate and Q20 were 0.03% and 97.51% in each sample, which indicates that the transcriptome data were reliable (Table S2). On average, 83.65% of the reads from *S. paraplesia* flowers matched the *S. purpurea* reference genome (Table S3). A total of 38534 genes were obtained, and the gene expression levels were similar among the different flowering stages (Fig. S1). Pearson correlation analysis revealed high similarity (R^2^ > 0.8) among biological replicates within the same group, which indicated that the data were suitable for transcriptome analysis (Fig. S2). PCA also indicated that the flower samples from the same group exhibited similar gene expression patterns. Flower samples of the same sex from different flowering stages were separated by PC1 (42.07%) and PC2 (15.40%). The male flower samples were more dispersive than the female flower samples, which means that gene expression patterns vary during flowering. In addition, flower samples from the different sexes could be distinctly separated by PC2, indicating that male and female flowers exhibited expression variation at the same stage (Fig. 4c). Hierarchical clustering analysis showed that male and female flowers exhibited more similar gene expression patterns in the early stage than in other stage. The samples could be divided into the same clusters as in the PCA, in which female flowers exhibited more similar gene expression patterns in the blooming and late flowering stages than in the early stage. Gene expression in male flowers in the late stage was the most different from that in the previous stages (Fig. 4a).

The number of sex-biased genes increased from 3751 to 9719 from the early to late stages (Fig. 4a, b). Male flowers exhibited more variation in gene expression than female flowers at all stages. We also found that only 4.70% of sex-biased genes exhibited sex-biased expression in all three stages. Of these, 48.3% of genes did not exhibit sex-biased expression in the first two stages, which indicated that the sex-biased genes were polytropical (Fig. 4e). Additionally, the number of male-biased genes was greater than that of female-biased genes in the first two stages (Fig. 4a). Furthermore, we investigated the expression patterns of 8157 genes that exhibited sex-biased expression in the late flowering stage but not in the early flowering stage. These genes were mainly downregulated in female catkins (1746 of 4039) and upregulated in male catkins (2345 of 4120) (Table 1).

**Table 1:** Variation in the expression of sex-biased genes in female and male catkins between the late (S3) and early (S1) flowering stages

Sex-biased pattern	Variation in female flowers	Variation in male flowers	Number of genes
Female-biased	Up	Up	106
	Down	Up	0
	Ns	Up	1
	Up	Down	48
	Down	Down	768
	Ns	Down	1746
	Up	Ns	599
	Down	Ns	7
	Ns	Ns	762
Male-biased	Up	Up	1077
	Down	Up	36
	Ns	Up	2345
	Up	Down	0
	Down	Down	5
	Ns	Down	2
	Up	Ns	25
	Down	Ns	108
	Ns	Ns	522

*Up*, upregulated genes; down, downregulated genes; *Ns*, no significant variation in gene expression between the late stage and the early stage

In addition, to confirm the reliability of the transcriptome data, we selected 19 sex-biased genes involved in different pathways in the three flowering stages for analysis with real-time quantitative PCR. The R^2^ values of the Pearson correlation coefficients were 0.95, 0.92, and 0.72 in the three flowering stages, which indicated that the RT-qPCR results were consistent with the transcriptome profile in each flowering stage (Fig. S4).

### Sex-biased genes located on chromosomes

We confirmed 13,593 DEGs between female and male flowers (*P*_*adj*_ < 0.05, |log_2_FoldChange | >1) but did not detect sex-limited gene expression. Furthermore, we found that sex-biased genes were more abundant on autosomes than on sex chromosomes. Compared with those of genes on autosomes, the number and proportion of sex-biased genes were lower on the putative sex chromosomes chr15Z and chr15W throughout the entire flowering stage. The autosome chr16 and the sex chromosome chr15w had the most and least sex-biased genes at each flowering stage, respectively (Fig. S3). Additionally, we also found that the number and proportion of sex-biased genes were higher on chr15Z than on chr15W.

### The function of sex-biased genes in flowers

GO analysis (*P*_*adj*_ < 0.05) was performed on the sex-biased DEGs at the different flowering stages (Table S5). The female-biased expressed genes involved in cell components were significantly enriched in the thylakoid, photosynthetic membrane, photosystem, and oxidoreductase complex pathways in the early and blooming flowering stages, while they were significantly enriched in the ribosome pathway in the late flowering stage. In terms of biological processes, female-biased genes were significantly enriched in photosynthesis in the early and blooming flowering stages and were enriched in translation, DNA replication, ribosome biogenesis, peptide metabolism, and amide metabolism in the late stage. Correspondingly, the male-biased genes involved in cell components were significantly enriched in the extracellular region in the blooming and late stages. In terms of biological processes, male-biased genes were significantly enriched in some negative-regulation processes in the early stage but were enriched in cellular carbohydrate and lipid metabolic processes, the cell wall, ion transport, and external encapsulating structural organization in the late stage.

**Fig. 5:**
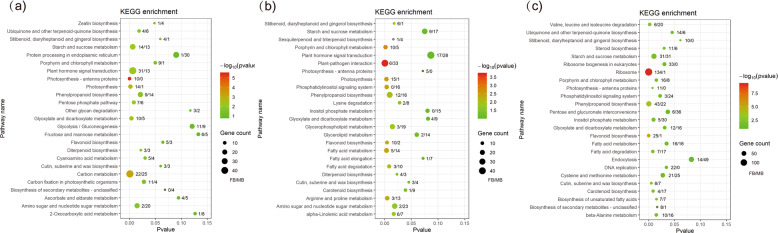
KEGG pathway enrichment of sex-biased genes in three flowering stages. **a** KEGG pathway enrichment of sex-biased genes in the early flowering stage. **b** KEGG pathway enrichment of sex-biased genes in the blooming flowering stage. **c** KEGG pathway enrichment of sex-biased genes in the late flowering stage. FB/MB represents the number of female-biased and male-biased genes. The size of the bubble indicates the number of sex-biased genes in the pathway; the color range represents the significance of pathway enrichment

There were 529, 634, and 1536 sex-biased genes that were annotated to 112, 115, and 117 KEGG pathways and significantly enriched (*P* < 0.05) in 15, 18, and 23 KEGG pathways in the three flowering stages, respectively (Fig. 5). The porphyrin and chlorophyll metabolism, carotenoid biosynthesis, and flavonoid biosynthesis pathways were all significantly enriched in the blooming and late flowering stages. These enriched pathways are associated with color development. In addition, at the early flowering stage, sex-biased genes were also significantly enriched in plant hormone signal transduction, as well as three pathways involved in photosynthesis and five pathways involved in carbohydrate metabolism. At the blooming stage, sex-biased genes were significantly enriched in plant–pathogen interactions, photosynthesis, fatty acid metabolism and degradation and secondary metabolism. At the late flowering stage, sex-biased genes were significantly enriched in ribosomes and DNA replication, which are associated with cell proliferation. All these enriched pathways are related to differences in growth and reproductive costs between male and female flowers.

### Metabolic differences between male and female flowers

**Fig. 6:**
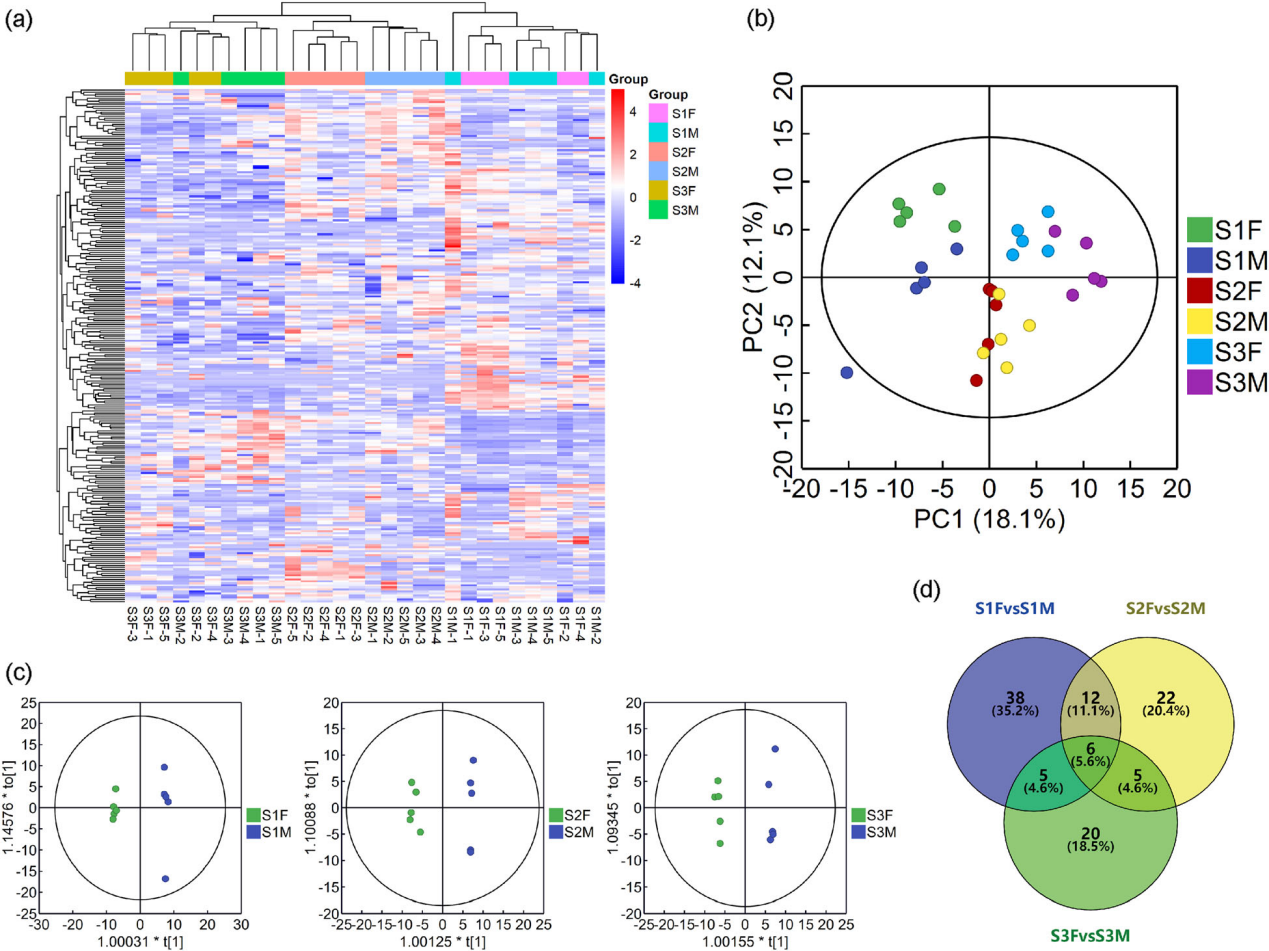
The metabolomic profiles of male and female *S. paraplesia* flowers. S1, early stage; S2, blooming stage; S3, late stage; F, female; M, male. **a** Bidirectional hierarchical clustering of identified metabolites and sample clustering analysis. The color palette represents the relative variation in the contents of metabolites among flower samples. Rows represent metabolites; columns represent flower samples. Red represents high gene expression; green represents low gene expression. **b** The score plot of the PCA of the metabolite profiles of the flower samples in the three flowering stages. PC1, first principal component; PC2, second principal component. **c** Score plot of the OPLS-DA analysis of metabolites in male and female flowers in the three flowering stages. X axis, T score; Y axis, orthogonal T score. **d** Venn diagram of differential metabolites in female and male flower samples in the three stages

**Fig. 7:**
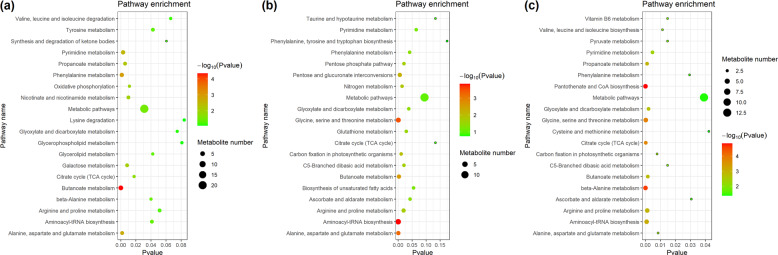
KEGG pathway enrichment of sexually differentiated metabolites in three flowering stages. **a** KEGG pathway enrichment of sexually differentiated metabolites in early flowering stage. **b** KEGG pathway enrichment of sexually differentiated metabolites in blooming flowering stage. **c** KEGG pathway enrichment of sexually differentiated metabolites in late flowering stage. The size of the bubble indicates the number of sexually differentiated metabolites in the pathway; the color range represents the significance of the pathway enrichment

A total of 256 metabolites were identified in the flower samples, and 216 metabolites were annotated to the KEGG database (Tables S6, S7). In the PCA, the flowering samples in the different stages could be separated by PC1 (18.10%) and PC2 (12.10%) (Fig. 6b). In addition, OPLS-DA analysis showed that the male and female flower samples in the same stage exhibited significantly different metabolic profiles (Fig. 6c). Furthermore, we identified 61, 45, and 36 metabolites whose contents were significantly different (VIP > 1 and *P* < 0.05) between male and female flowers in the three stages (Table S8). The contents of only 5.6% of the sexually differentiated metabolites exhibited sexual difference during all three flowering stages, which indicated that sexual dimorphism in the flowers varied with flower development (Fig. 6d). Interestingly, most of the sexually differentiated metabolites identified during the different stages were enriched in the same pathways, such as butanoate metabolism and alanine, aspartate, and glutamate metabolism (Fig. 7).

## Discussion

**Fig. 8:**
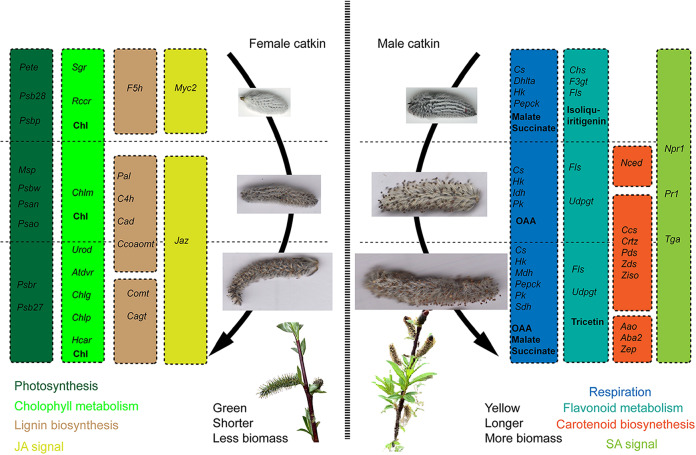
Schematic of sexual dimorphism in *S. paraplesia* flowers during florescence. Pathways on the left are dominated by female-biased genes. Pathways on the right are dominated by male-biased genes. Gene abbreviations: *Aao*, abscisic-aldehyde oxidase; *Aba2*, xanthoxin dehydrogenase; *Atdvr*, divinyl chlorophyllide an 8-vinyl-reductase; *C4 h*, trans-cinnamate 4-monooxygenase; *Cad*, cinnamyl-alcohol dehydrogenase; *Cagt*, coniferyl-alcohol glucosyltransferase; *Ccoaomt*, caffeoyl-CoA O-methyltransferase; *Ccs*, capsanthin/capsorubin synthase; *Chlg*, chlorophyll synthase; *Chlm*, magnesium protoporphyrin IX methyltransferase; *Chlp*, geranylgeranyl diphosphate reductase; *Chs*, chalcone synthase; *Comt*, catechol O-methyltransferase; *Crtz*, beta-carotene 3-hydroxylase; *Cs*, citrate synthase; *Dhlta*, dihydrolipoyllysine-residue acetyltransferase; *F3 gt*, anthocyanidin 3-O-glucosyltransferase; *F5 h*, ferulate-5-hydroxylase; *Fls*, flavonol synthase; *Hcar*, 7-hydroxymethyl chlorophyll a reductase; *Hk*, hexokinase; *Idh*, isocitrate dehydrogenase; *Jaz*, jasmonate ZIM domain-containing protein; *Mdh*, malate dehydrogenase; *Msp*, manganese-stabilizing protein; *Myc2*, transcription factor MYC2; *Nced*, 9-cis-epoxycarotenoid dioxygenase; *Npr1*, regulatory protein NPR1; *Pal*, phenylalanine ammonia-lyase; *Pds*, 15-cis-phytoene desaturase; *Pepeck*, phosphoenolpyruvate carboxykinase (ATP); *Pete*, plastocyanin; *Pk*, pyruvate kinase; *Pr1*,pathogenesis-related protein 1; *Psan*, photosystem I subunit PsaN; *Psao*, photosystem I subunit PsaO; *Psb27*, photosystem II Psb27 protein; *Psb28*, photosystem II 13 kDa protein; *Psbp*, photosystem II oxygen-evolving enhancer protein 2; *Psbr*, photosystem II 10 kDa protein; *Psbw*, photosystem II PsbW protein; *Rccr*, red chlorophyll catabolite reductase; *Sdh*, succinate dehydrogenase; *Sgr*, magnesium dechelatase; *Tga*, transcription factor TGA; *Udpgt*, UDP-glycosyltransferase; *Urod*, uroporphyrinogen decarboxylase; *Zds*, zeta-carotene desaturase; *Zep*, zeaxanthin epoxidase; *Ziso*, 15-cis-zeta-carotene isomerase. Metabolite abbreviations: Chl, chlorophyll; OAA, oxaloacetic acid

Sexual dimorphism in flowers evolved as a result of the overall fitness gains associated with having separate male and female structures and different reproductive roles during mating. Our study found that there was significant dimorphism between male and female *S. paraplesia* flowers at the phenotypic, metabolic, and transcriptional levels during florescence. Compared with female flowers, the male flowers of *S. paraplesia* exhibited more sex-biased gene expression. The differences in the gene expression and metabolic profiles of *S. paraplesia* flowers not only are responsible for the primary sexual dimorphism, *i.e*., the androecium in males and the gynoecium in females, but also contribute to the secondary sexual dimorphism of flowers in terms of their morphology and growth, energy consumption and catkin color during development (Figs. 8, S5–S7).

### Dimorphism in flower growth and stress resistance

Considering the different reproductive roles of unisexual flowers, dioecious plants usually make different reproductive investments in male and female individuals. In our study, the male catkins were larger and longer than the female catkins at the three flowering stages. This probably occurs because *S. paraplesia* is a wind-pollinated dioecious species and tends to produce excess pollen to increase its mating success rate, which leads to male catkins of *S. paraplesia* elongating their rachis and generating more stamens with long pollen tubes during early florescence. In contrast, female catkins did not generate stigma until later fluorescence, before pollination. Therefore, male catkins have a higher reproductive cost for growth than females at earlier fluorescence times. We also found that male catkins exhibited greater dry mass and higher C, N, and P contents than females per catkin, which indicated that male catkins obtained greater reproductive investments than females from vegetative organs.

On the other hand, this sexual dimorphism in catkin morphology and biomass may be associated with sexual dimorphism during flowering that is related to phytohormones. Auxin, gibberellins (GAs), and brassinosteroids are closely related to flower bud growth and development^53–55^. GAs are essential regulators of plant growth in terms of stem elongation and flower development^56–58^. We found that sex-biased genes were enriched in the GA and auxin signaling pathways. Plants can regulate downstream gene expression *via* the GA signaling pathway^59,60^. It is worth noting that we found that a DELLA protein family gene (*Sapur.010G083400*) showed female-biased expression at all three flowering stages, while another *Della* gene (*Sapur.017G103500*) was male-biased at the late flowering stage (Table S9). The DELLA protein is a crucial negatively regulated protein in the GA signaling pathway, and the expression of *Della* genes is repressed by GAs^61^. In our study, the female-biased *Della* gene may have caused the male catkins to exhibit lower levels of the *Della* protein, which led to male catkins being more sensitive to GA signals at earlier fluorescence times. In this scenario, male catkins would need less GA to stimulate the development of the stamen. Additionally, the dimorphism of gene expression might partially explain why the rachis of male catkins was longer than that of females.

This sexual dimorphism in catkin growth also exhibited temporal dynamics, which may correlate with the different reproductive roles of male and female *S. paraplesia* flowers. At the late stage, before pollination, female flowers generate stigma to receive pollen, while male flowers achieve mature functions and reproductive structures; this difference leads to sexual dimorphism in cell growth and division in catkins. DNA replication and ribosome biogenesis are essential processes in mitosis and meiosis, and genes involved in these processes can regulate cell proliferation^62–65^. The transcriptome profiles showed that sex-biased genes were enriched in DNA replication and ribosomes and were dominated by female-biased genes at the late flowering stage (Fig. 5). The majority of those genes exhibited nonbiased expression at the early and blooming stages; however, they were downregulated in male flowers but upregulated in female flowers in the late stage, showing significantly female-biased expression. These sex-biased genes and the temporal changes in their expression reflected that female catkins exhibited stronger cell proliferation than males at late stages before pollination. This phenomenon may be ascribed to male catkins reducing cell proliferation and growth after pollen generation, while female catkins generate stigma at the late flowering stage. These female-biased genes lead to morphological differences between male and female flowers in the late flowering stage and are responsible for the longer catkin after pollination in female flowers.

In addition to the sexual differences in growth, sexual dimorphism is also reflected in flower stress resistance. Salicylic acid (SA) and jasmonic acid (JA) play important roles in the plant response to biotic and abiotic stresses, and these signaling pathways are complex networks of synergetic and antagonistic effects^66–70^. In our study, the female-biased (*MYC2*) and male-biased genes (*JAZ*) involved in JA signaling exhibited positive and negative regulatory effects, respectively. In the SA signaling pathway, the male-biased genes exhibited positive regulatory effects (Table S9). This indicated that male flowers of *S. paraplesia* were more sensitive to SA signaling, while female flowers were more sensitive to JA signaling Therefore, we believe that the sexual dimorphism in the SA and JA signaling pathways may, on the one hand, reflect their antagonistic effects^71,72^ and, on the other hand, reflect the different defensive strategies of the sexes. Additionally, in the plant–pathogen interaction pathway, sex-biased genes that encode the proteins of CaM/CML, CNGCs, and WRKY33 might also cause sexual dimorphism in response to pathogen and bacterial damage in *S. paraplesia* flowers.

### Dimorphism in flower energy consumption

**Fig. 9:**
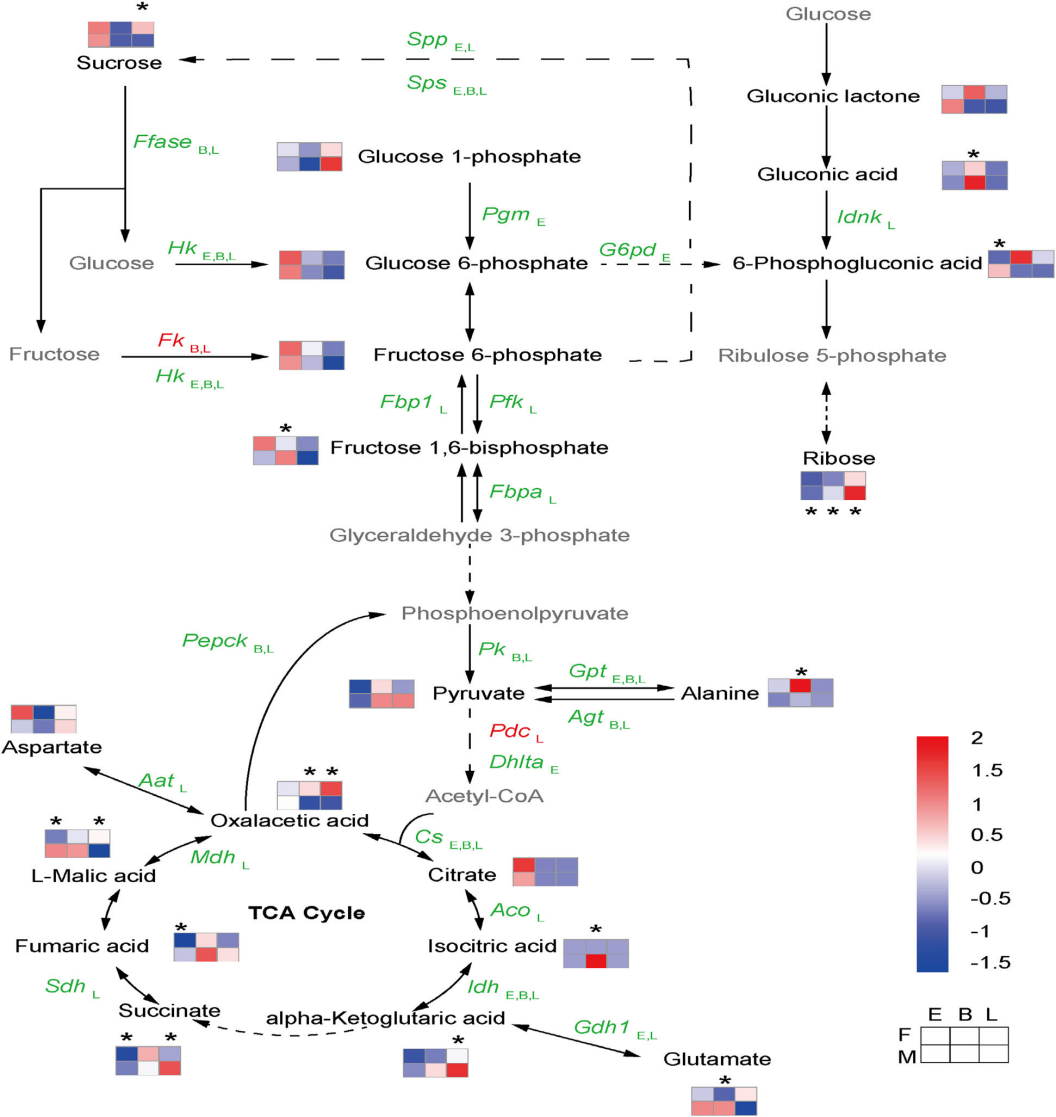
Sexual dimorphism in respiration in *S. paraplesia* flowers in three flowering stages. The gray color represents undetected metabolites. The squares and the color palette represent the relative content of metabolites in the three flowering stages. The asterisk represents *p* values of less than 0.05 according to a *t*-test. The italic text represents sex-biased genes. The red and green colors represent female-biased and male-biased expression, respectively. F, female; M, male; E, early-stage; B, blooming stage; L, late-stage; Gene abbreviations: *Aat*, aspartate aminotransferase; *Acl*, ATP citrate synthase; *Aco*, aconitate hydratase; *Agt*, alanine-glyoxylate transaminase; *Cs*, citrate synthase; *Dhlta*, dihydrolipoyllysine-residue acetyltransferase; *Fbp1*, fructose-1,6-bisphosphatase; *Fbpa*, fructose-bisphosphate aldolase; *Ffase*, beta-fructofuranosidase;*Fk*, fructokinase; *G6pd*, glucose-6-phosphate dehydrogenase; *Gdh1*, glutamate dehydrogenase; *Hk*, hexokinase; *Idh*, isocitrate dehydrogenase; *Idnk*, gluconokinase; *Mdh*, malate dehydrogenase; *Pdc*, pyruvate decarboxylase; *Pepck*, phosphoenolpyruvate carboxykinase (ATP); *Pfk*, 6-phosphofructokinase; *Pgm*, phosphoglucomutase; *Pk*, pyruvate kinase; *Sdh*, succinate dehydrogenase; *Spp*, sucrose-6-phosphatase; *Sps*, sucrose-phosphate synthase

Dioecious plants usually exhibit differential reproductive investment in male and female individuals. In our study, in addition to their greater biomass and C content, male flowers of *S. paraplesia* also exhibited higher C metabolism than female flowers before pollination. As the main pathways for cellular respiration, glycolysis, the pentose phosphate pathway, and the tricarboxylic acid cycle (TCA cycle) are central carbohydrate metabolism pathways of cell energy and material metabolism^73–75^. We found that female flowers had higher sucrose levels than male flower in the late flowering stage. Considering the predominance of male-biased genes involved in the conversion of sucrose to hexose and the glycolysis pathway, we believe that male flowers exhibit greater carbohydrate consumption than females during flower development (Fig. 9).

Furthermore, the TCA cycle was also dominated by male-biased genes, such as the genes that encode citrate synthase, aconitate hydratase, isocitrate dehydrogenase, succinate dehydrogenase, and malate dehydrogenase (Fig. 9). Additionally, male-biased metabolites, such as isocitric acid, ketoglutaric acid, and succinate, were involved in the TCA cycle in all three flowering stages. These changes in gene expression and metabolites suggested that male flowers exhibited greater TCA cycling than females. Interestingly, we found that the genes encoding phosphoenolpyruvate carboxykinase (PEPCK) were male-biased and might accelerate the production of phosphoenolpyruvic acid (PEP) from oxaloacetic acid (OAA). Previous studies have indicated that PEPCK plays an important role in the TCA cycle and glycolysis and affects the sugar contents of plant organs^76–78^. PEPCK controls the first step of gluconeogenesis, where glucose is synthesized from malic acid and OAA or noncarbohydrate carbon substrates such as asparagine, aspartate, alanine, and glutamine^79–81^. Osorio et al*.*^82^ found that the inhibition of PEPCK expression could result in the accumulation of malic acid and decrease sugar and starch levels in tomato fruits. In our study, we found that male flowers enhanced gluconeogenesis from malate to produce more PEP and that PEP could reenter mitochondria and participate in the TCA cycle. Our results suggested that male flowers of *S. paraplesia* exhibited greater respiration flux than female flowers due to their higher gene expression and metabolite levels. In *Populus balsamifera*, respiration in flowers is also dominated by male-biased gene expression^20^. These greater glycolysis and TCA cycle activities indicated that male flowers of *S. paraplesia* enhanced their energy metabolism to provide more energy and critical molecules within the core of the metabolic network. As critical reproductive tissues, the stamen and stigma are completely heterotrophic and rely on carbon source tissues. Owing to their different roles in mating, the generation of stamens in male flowers usually occurs earlier than the generation of stigma in female flowers of *S. paraplesia*. As a result, male flowers demand more energy from respiration to produce stamens and pollen, while female flowers consume less energy and store carbohydrates for fruition after pollination. Even so, male flowers still have more biomass and higher C contents, suggesting that the reproductive cost per male flower is far higher than that per female flower before pollination.

### Dimorphism in flower pigment biosynthesis

*Paraplesia* is a wind-pollinated species, and it does not need to produce colorful male flowers to attract pollinators. However, sexual dimorphism in catkin color was exhibited in the last two flowering stages. We observed yellow or red stamens in male catkins, while the female catkins were green. Similar to our results, Sanderson et al*.*^20^ found that female flowers were green and remained photosynthetically active through the stages of flowering. The color of flowers is determined by pigments such as chlorophyll, carotenoids, and flavonoids^83,84^. Unsurprisingly, porphyrin and chlorophyll metabolism were significantly enriched at all flowering stages and were dominated by female-biased genes; carotenoid biosynthesis was significantly enriched in the last two flowering stages and was dominated by male-biased genes. Compared with male flowers, female flowers of *S. paraplesia* exhibited higher chlorophyll contents in the three flowering stages (Fig. S9). As the essential phytochromes in plants, chlorophyll contributes to the green color of plant tissues, and carotenoids contribute to their red, yellow, and orange colors^85–88^. On the other hand, *Salix paraplesia* develops flowers before developing leaves in high-altitude, low-temperature habitats, and the high chlorophyll concentrations in female catkins allow them to maintain a certain level of photosynthesis to meet the high C demand before leaf germination.

Secondary metabolites, *e.g*., terpenes, phenols, and alkaloids, play important roles in growth, reproduction, and stress resistance in plants^89,90^. Flavonoid biosynthesis, as the downstream pathway of phenylpropanoid biosynthesis, was enriched in the last two flowering stages. Flavonoids play multiple roles in plants and affect antioxidant activity, stress resistance, pollen germination, and flower color^91,92^. Altitude can influence the flavonoid contents of plants on plateaus^93^. The high UV-B radiation at higher altitudes can cause damage to plant cells^94,95^. For *S. paraplesia* growing in alpine habitats, an increase in epidermal flavonoid content could not only alleviate photoinhibition but also protect plants from oxidative damage caused by high UV-B radiation^96,97^. At the early and blooming stages of flowering, male-biased genes dominated the metabolism of several flavonoids, including the biosynthesis of chalcones, isoflavonoids, and flavonols (Fig. S8). Metabolically, we also detected that male flowers exhibited higher isoliquiritigenin and tricetin levels than females (Table S8). In contrast, female-biased genes dominated the biosynthesis of chalcones, isoflavonoids, and dihydroflavonols in the late flowering stage. The predominant male-biased genes involved in flavonoid metabolism in the first two flowering stages and the higher phenol content contribute to sexual dimorphism with regard to resistance to UV-B radiation, disease, and insects; the constitution of the stamen; and the pollen color of male flowers.

We believe that these sex-biased genes are related to the observed phenotypic differences, *i.e*., female flowers of *S. paraplesia* are more green, while males are more yellow. On the other hand, considering the dominant female-biased genes in the photosynthesis pathway, which are consistent with their higher chlorophyll contents, female catkins can exhibit higher in situ photosynthetic activity than male catkins. This sexual dimorphism in the genes related to plant pigments in *S. paraplesia* flowers is similar to the case of *P. balsamifera*, in which the female catkins are greener and more photosynthetically active than the male catkins^20^. Considering that *S. paraplesia* is a wind-pollinated species, sexual dimorphism in flower color might not directly influence pollination or mating.

In conclusion, we determined that a large number of sex-biased genes, rather than sex-limited genes, cause sexual dimorphism in *S. paraplesia* flowers and that they are dynamic, changing throughout the stages of flowering. This temporal dynamic is caused mainly by sex-biased gene expression in male flowers during florescence. Considering the biomass, nutrient, and morphological observations from this study, we believe that the sex-biased genes and metabolites well explain the sexual dimorphism in the color, growth, and reproductive cost of male and female catkins. As we expected, more carbohydrates are allocated to individual male flowers of *S. paraplesia* than to female flowers in order to meet their greater biomass and higher energy consumption requirements before pollination. Therefore, our results demonstrate for the first time that sex-related differential reproductive investment occurs in *S. paraplesia* flowers at the molecular level.

## Supplementary information

Supplemental Fig S1-9

Supplemental Table S1

Supplemental Table S2

Supplemental Table S3

Supplemental Table S4

Supplemental Table S5

Supplemental Table S6

Supplemental Table S7

Supplemental Table S8

Supplemental Table S9
