# Supplementary material for: Sex-biased genes and metabolites explain morphologically sexual dimorphism and reproductive costs in Salix paraplesia catkins
Source: Hortic Res. 2021 Jun 1;8:125. doi: 10.1038/s41438-021-00566-3 (PMC8166972; doi:10.1038/s41438-021-00566-3)

Supplement Fig. S1 Gene expression distribution in each flower samples. S1, early stage; S2, blooming stage; S3, later stage; F, female; M, male.

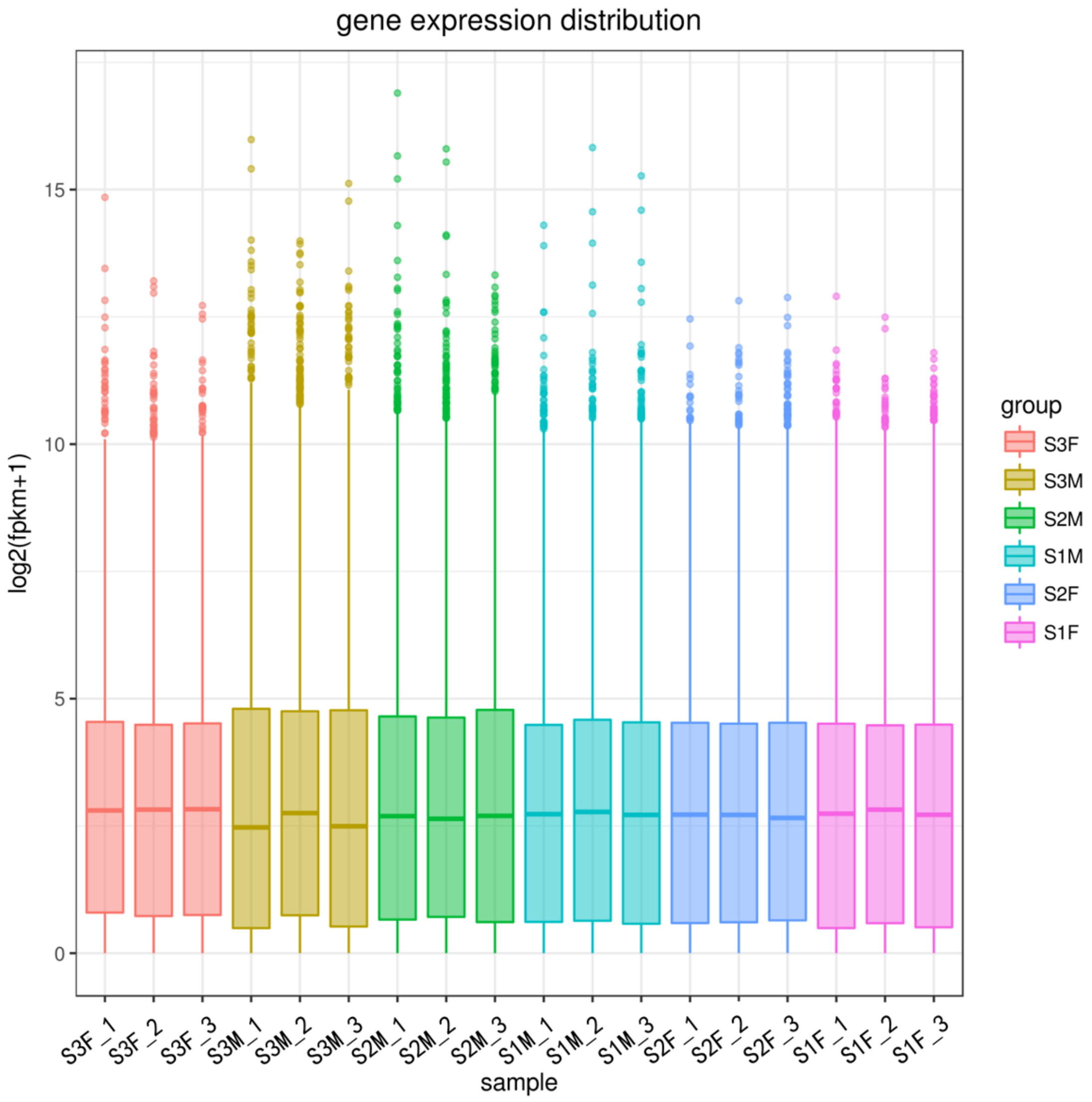

Supplement Fig. S2 The correlation of gene expression pattern between samples. R^2, square of Pearson Correlation Coefficient. S1, early stage; S2, blooming stage; S3, later stage; F, female; M, male.

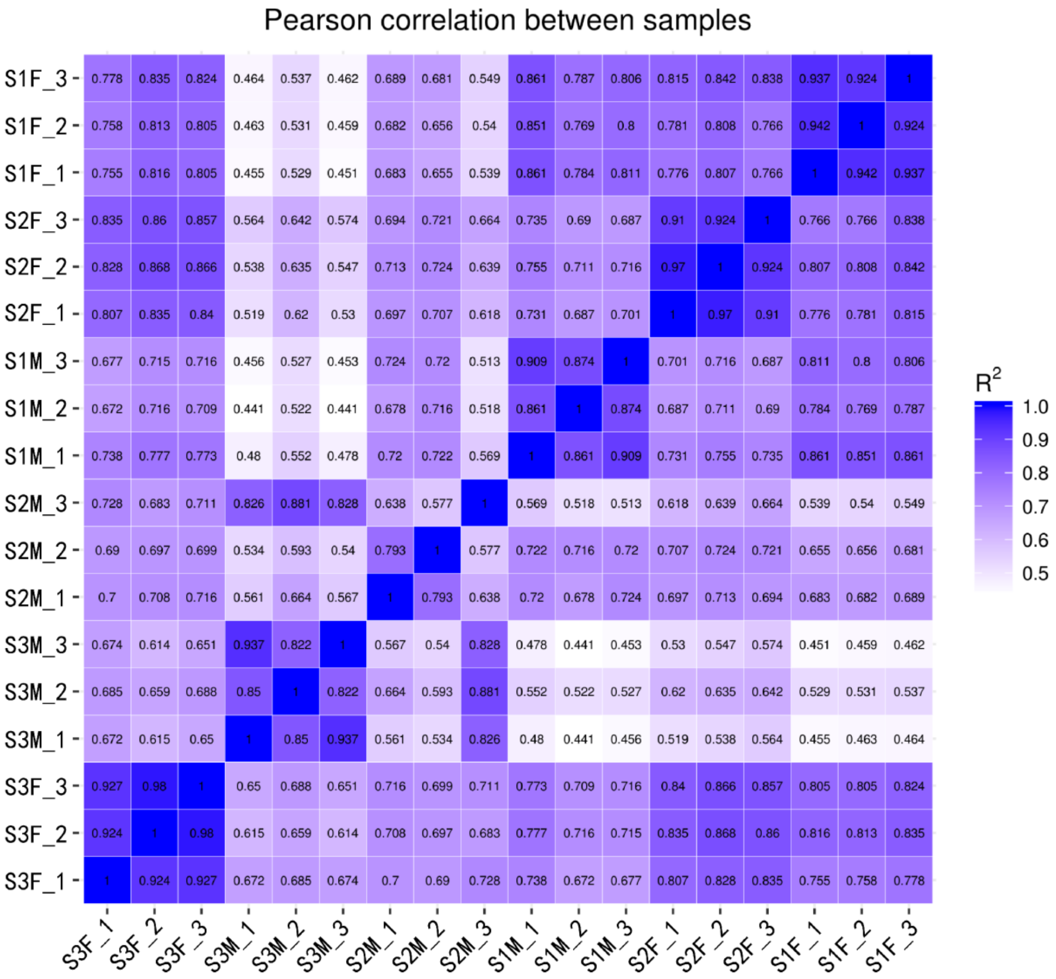

Supplement Fig. S3 The chromosome location of sex-biased genes. Black columns represent the number of sex-biased genes, blue points represent the ratio of sex-biased genes numbers to all genes in chromosome.

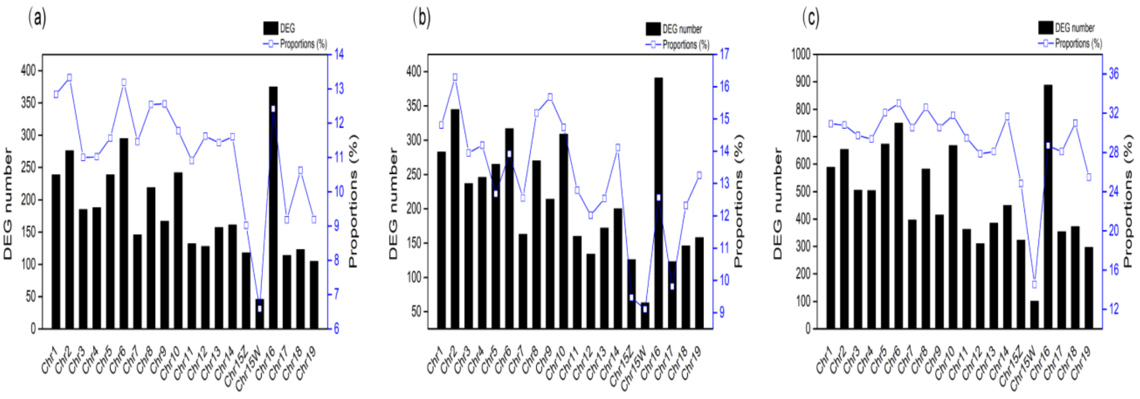

Supplement Fig. S4 The validation of real-time qPCR on sex-biased genes of *S. paraplesia* flower samples in early stage (a), blooming stage (b) and later stage (c).

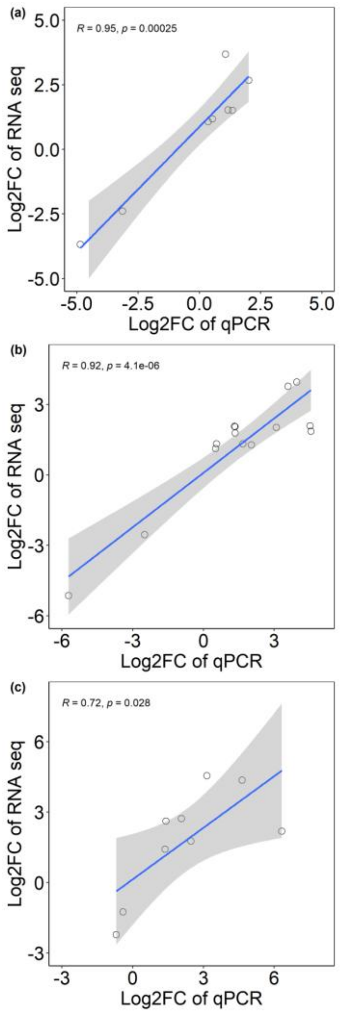

Supplement Fig. S5 Metabolic pathways of sex-biased genes in male and female flowers of *S. paraplesia* in early stage. Each square represents single sex-biased gene. The color palette represent the Log_2 (fold change) of genes. Red indicats female-biased expression; blue indicats male-biased expression. EMP, glycolytic pathway; Minor CHO, minor carbohydrate metabolism; OPP, oxidative pentose phosphate pathway; TCA, tricaboxylic acid cycle.

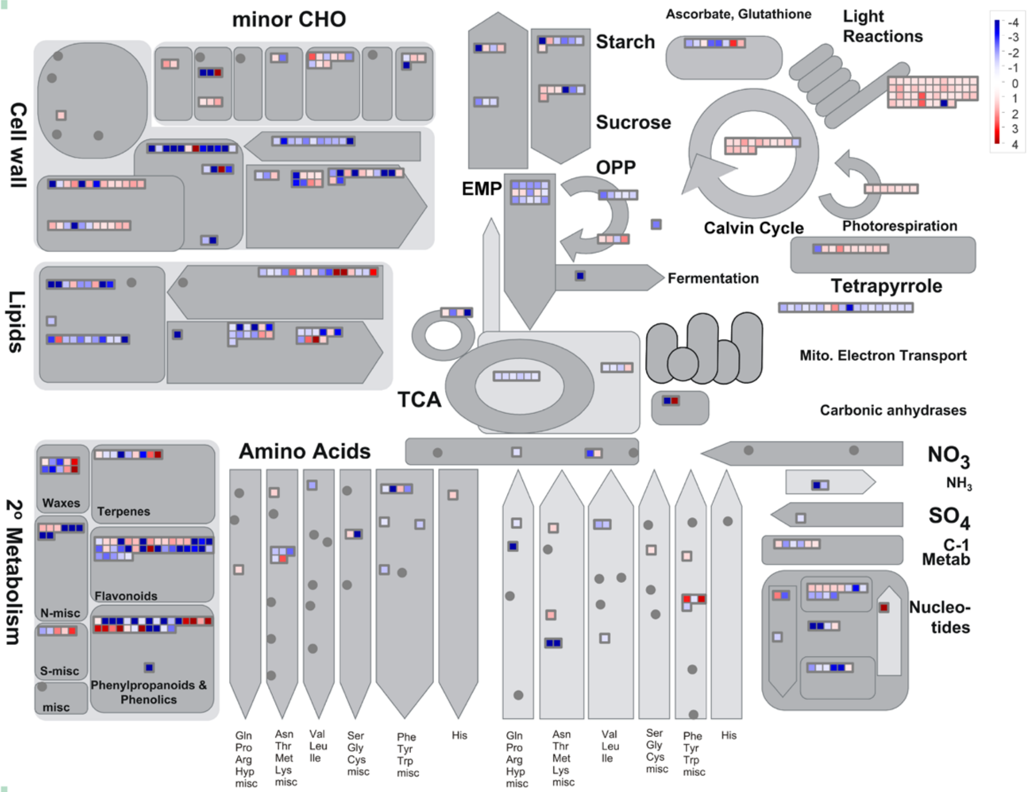

Supplement Fig. S6 Metabolic pathways of sex-biased genes in male and female flowers of *S. paraplesia* in blooming stage. Each square represents single sex-biased gene. The color palette represent the Log_2 (fold change) of genes. Red indicats female-biased expression; blue indicats male-biased expression. EMP, glycolytic pathway; Minor CHO, minor carbohydrate metabolism; OPP, oxidative pentose phosphate pathway; TCA, tricaboxylic acid cycle.

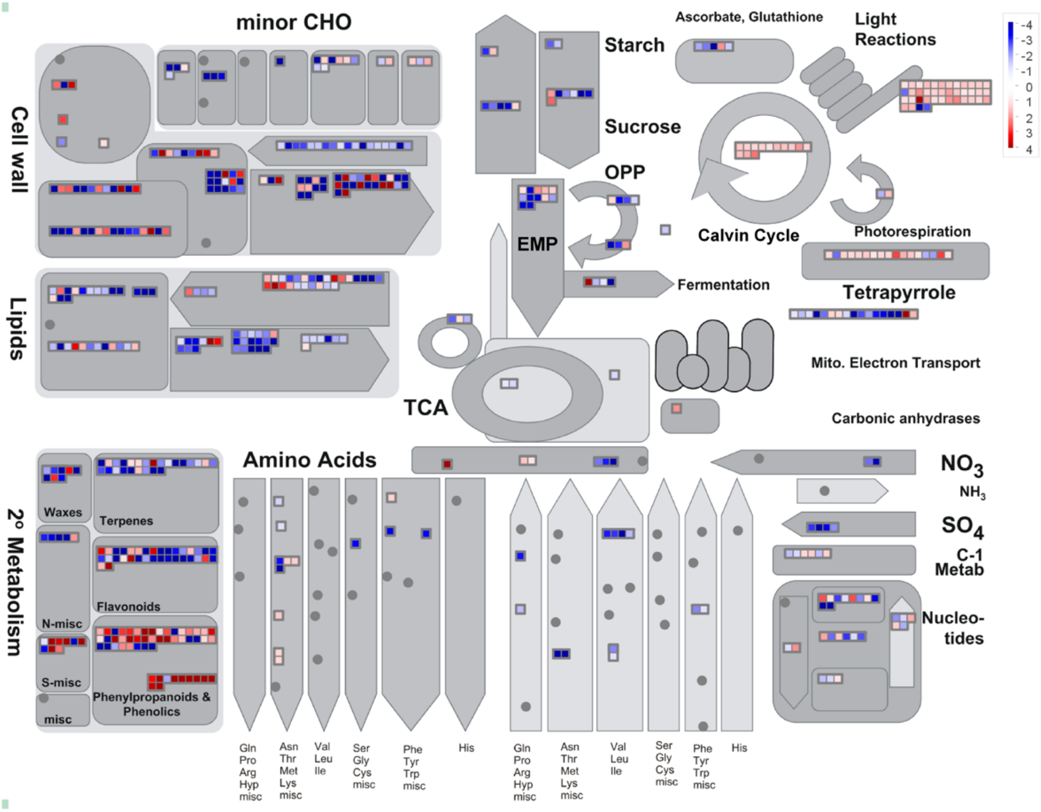

Supplement Fig. S7 Metabolic pathways of sex-biased genes in male and female flowers of *S. paraplesia* in later stage. Each square represents single sex-biased gene. The color palette represent the Log_2 (fold change) of genes. Red indicats female-biased expression; blue indicats male-biased expression. EMP, glycolytic pathway; Minor CHO, minor carbohydrate metabolism; OPP, oxidative pentose phosphate pathway; TCA, tricaboxylic acid cycle.

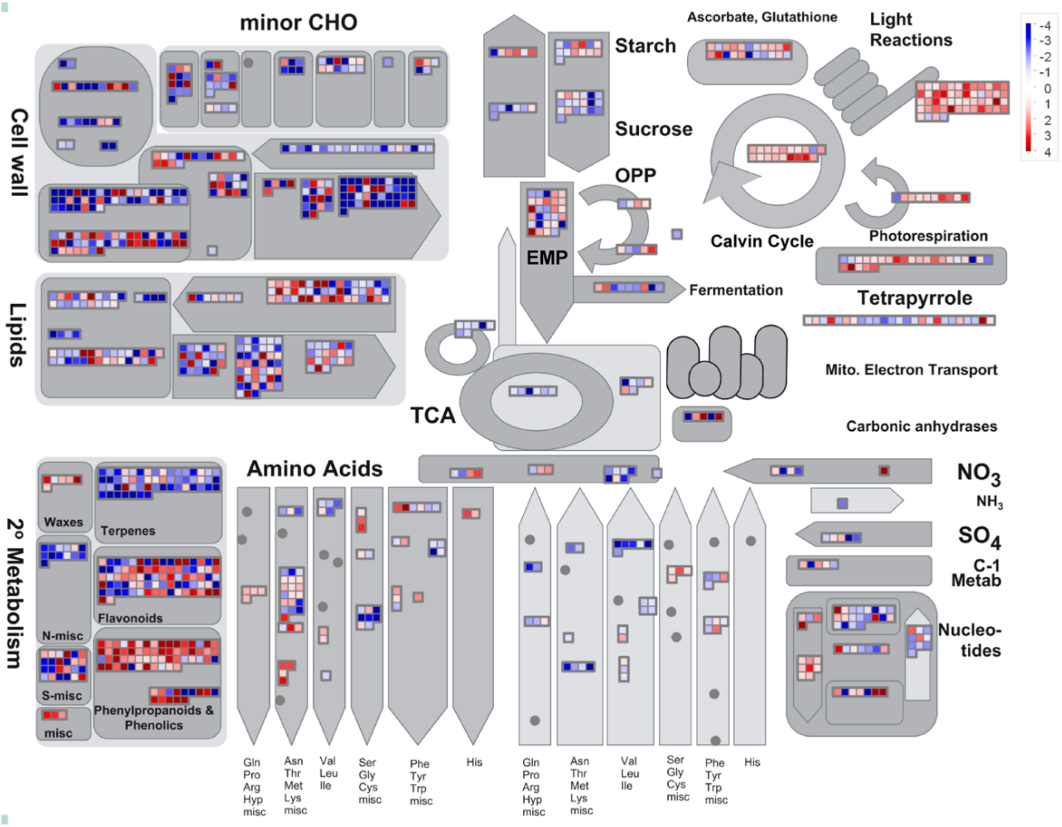

Supplement Fig. S8 Secondary metabolic pathways of sex-biased genes in male and female flowers of *S. paraplesia* in early (a), blooming (b) and later (c) flowering stages. Each square represents single sex-biased gene. The color palette represent the Log_2 (fold change) of genes. Red indicats female-biased expression; blue indicats male-biased expression.

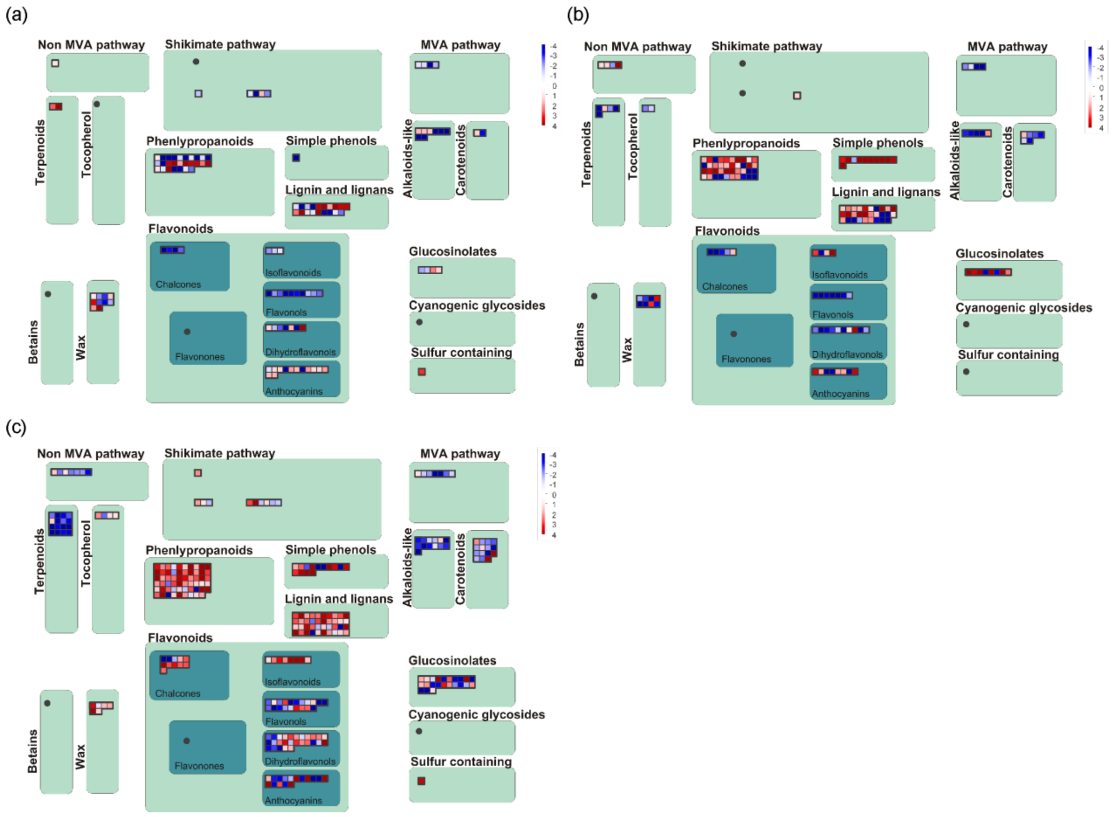

Supplement Fig. S9 Chlorophyll contents of male and female flowers of *S. paraplesia* in three flowering stages. Chla, chlorophyll a; Chlb, chlorophyll b; Chl, Chla+Chlb. Four asterisk (****) indicated *p* values of ANOVA (Tukey test method) less than 0.0001. Each value represents the mean ± SE (n=5).

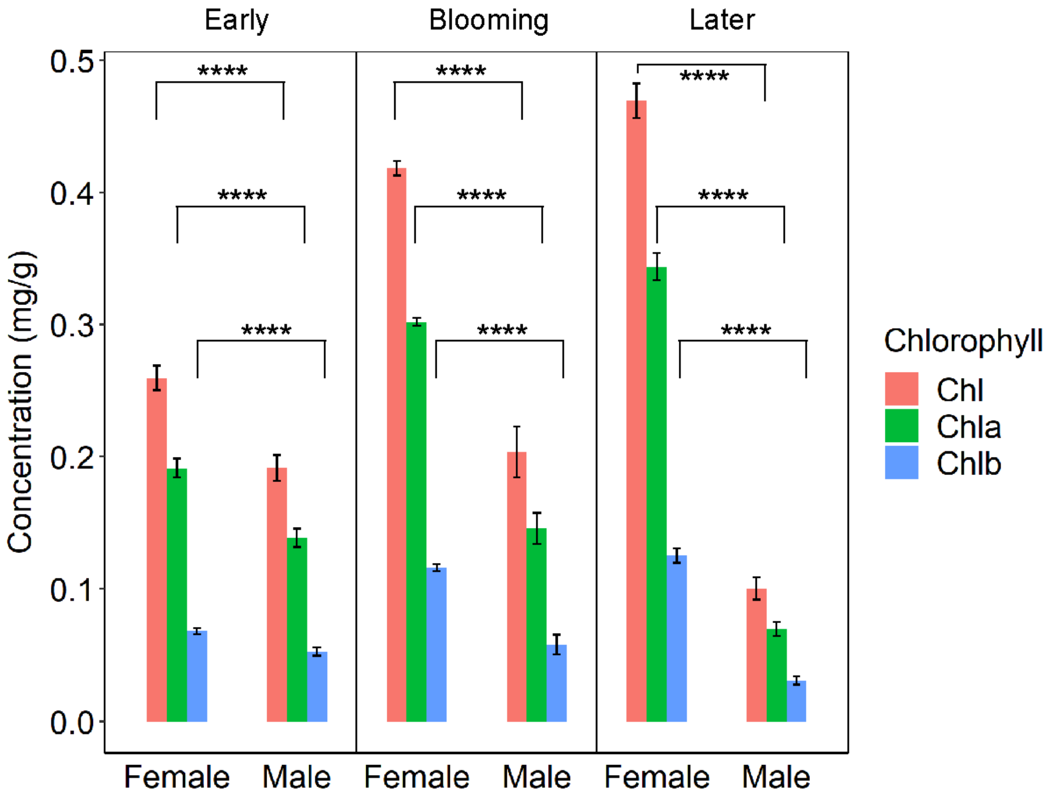

Supplement: Supplementary file 1 — Supplemental Fig S1-9 [file 41438_2021_566_MOESM1_ESM.pdf]
